# Tinnitus Abnormal Brain Region Detection Based on Dynamic Causal Modeling and Exponential Ranking

**DOI:** 10.1155/2018/8656975

**Published:** 2018-07-09

**Authors:** Ming-Chuan Tsai, Yue-Xin Cai, Chang-Dong Wang, Yi-Qing Zheng, Jia-Ling Ou, Yan-Hong Chen

**Affiliations:** ^1^School of Data and Computer Science, Sun Yat-sen University, Guangzhou, China; ^2^Department of Otolaryngology, Sun Yat-sen Memorial Hospital, Sun Yat-sen University, Guangzhou, China; ^3^Institute of Hearing and Speech-Language Science, Sun Yat-sen University, Guangzhou, China; ^4^Zhongshan School of Medicine, Sun Yat-sen University, Guangzhou, China

## Abstract

Dynamic Causal Modeling (DCM) has been extended for the analysis of electroencephalography (EEG) based on a specific biophysical and neurobiological generative model for EEG. Comparing to methods that summarize neural activities with linear relationships, the generative model enables DCM to better describe how signals are generated and better reveal the underlying mechanism of the activities occurring in human brains. Since DCM provides us with an approach to the effective connectivity between brain areas, with exponential ranking, the abnormality of the observed signals can be further located to a specific brain region. In this paper, a combination of DCM and exponential ranking is proposed as a new method aiming at searching for the abnormal brain regions which are associated with chronic tinnitus.

## 1. Introduction

Subjective tinnitus as a common clinical symptom is defined as a perceived sound in the absence of a corresponding internal or external sound source [1]. According to the duration of occurrence, tinnitus is classified into acute tinnitus and chronic tinnitus. People with acute tinnitus experience a transient and reversible perception of a phantom sound within 3 months, which has rather little impact on the life of people. And chronic tinnitus can last more than 3 months [2], making patients with chronic tinnitus suffer from insomnia, poor concentration, depression, and cognitive dysfunction. It is said that 10-15 % of the adult population suffers from chronic tinnitus and about 1-2% of the adult population is affected by the phantom sound severely [3].

Till now, the main clinical treatments of tinnitus include counseling, sound therapy, cognitive behavioral therapy, and tinnitus retraining therapy. These treatments vary greatly in the therapeutic effect and there is still a lack of medical, neurological, or neuropsychological therapy that has been proved as the universal treatment [4]. The effective treatment of tinnitus is based on the comprehensive understanding of the underlying mechanism of tinnitus. Therefore, to treat tinnitus, there is urgent need of understanding the generation and development of tinnitus.

Tinnitus has traditionally been considered as problems with the cochlea and the auditory nerves. Tinnitus patients are always accompanied by peripheral hearing loss [5], suggesting that the decreased signal output from the cochlea is associated with the generation of tinnitus. However, tinnitus still exists after cutting off the auditory nerve [6, 7], which indicates that tinnitus is not only caused by peripheral hearing loss, but also the result of aberrant neural activity of central nervous system. It is widely accepted that abnormalities in the central nervous system (CNS) play an important role in the development of tinnitus. In other words, it is the abnormal activation of brain regions, not just the auditory system that relate to tinnitus, which is the theoretical basis of this research.

A variety of methods for exploring brain activity exist and could provide different kinds of signals that sketch the brain activities, i.e., fMRI, EEG, and MEG. Many of the previous researches are based on the resting-state fMRI. The resting-state fMRI has been proved to be a useful noninvasive technique for determining how structurally segregated and functionally specialized cerebral centers are interconnected for subjective tinnitus [9]. However, due to the constant emission of detrimental scanner noise and other uncomfortable aspects of scanning environment, many patients refuse to participate in fMRI studies. In addition, EEG signals contain more information of the underlying dynamics than fMRI. Moreover, spectral power and connectivity analysis of the resting-state EEG have been turned out as advantageous tools because EEG parameters obtained from patients generally differ from EEG patterns of people without chronic tinnitus symptoms [10]. So EEG is considered as a more suitable technique to explore the functional signature of tinnitus [11].

**Figure 1:**
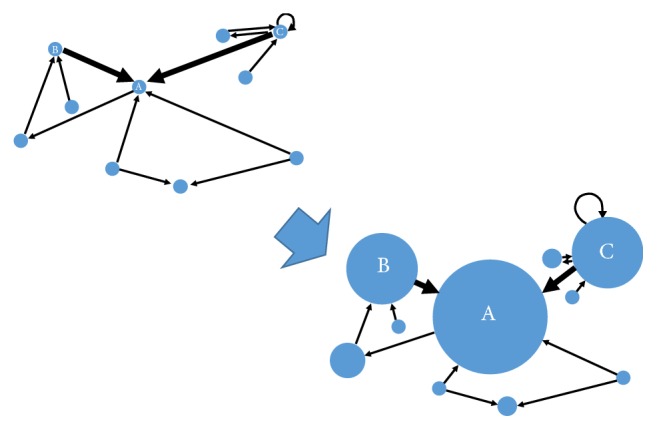
The process of PageRank-like algorithms.

Efforts have been made to generate brain connectivity from biological signals, with which they use current community detection and clustering algorithms [12–15] to reach their goal, such as research on Alzheimer's disease [16]. For tinnitus, several models have been proposed suggesting that abnormalities within nonauditory and between nonauditory and auditory brain regions and networks are associated with tinnitus [17–19]. These studies take the similarities of the signals observed as the connectivity between sensors. However, signals detected by the sensors are generated by wide-spread brain sources, which often contain lots of noise and greatly differ between two individuals, in spite of their conditions. Besides, the calculation of the similarity does not solve the “spatiotemporal inverse problem”, i.e., which brain regions caused the observed spatial and temporal pattern in the sensors [20]. In fact, for EEG analysis, classical methods usually try to reduce the temporal details and ignore the underlying generators. For instance, many researches characterize participants by the average of EEG over temporal windows, such as the power spectral estimation [21], the autoregressive (AR) model [22], and the power spectral entropy [23]. They may have good performance on nonlinear dynamic states but ignore the sequences of time and the causalities of brain sources. Dynamic causal modeling (DCM) is a candidate designed to solve this problem and to make inferences about key neuronal parameters based on spatiotemporal models [8]. It not only combines the dynamics and nonlinearity of the nervous system, but also organically combines the actual observation of the different brain signals (e.g., fMRI, MEG, and EEG) and the nerve level dynamics. In particular, the present study aims to find the abnormal brain regions that are associated with tinnitus. And abnormal brain regions are needed for the special treatment of tinnitus. To this end, we ought to find the abnormal brain regions that significantly distinguish tinnitus patients from the normal controls. Given the connectivity matrices gained from the previous DCM process, the task can be transferred into ranking each brain region and then investigating the ranking differences between the tinnitus patients and the normal controls. One suitable technique for achieving this task uses a PageRank-like method, which estimates the ranking values according to the weighted linkage structure (i.e., a weighted connectivity matrix) [24]. In the ranking algorithms, trust probabilities of the nodes are focused. By iteratively multiplying the adjacency matrix and the nodes' trust probabilities till convergence, the nodes in a network represented by its adjacency matrix can be ranked, as illustrated in Figure 1. In the sorting algorithm, we focus on the trust probability of the node, which is equivalent to the importance of brain regions in the brain network in this article, meaning that the higher a node ranks, the more activated it is. However, the original PageRank algorithm [24] is only applicable to the positive weighted links and therefore is not suitable for the case. Hence, the variant of PageRank, termed exponential ranking [25], is utilized, which is able to find the ranking of each brain region in the brain network. By further investigating the ranking differences between the tinnitus patients and the normal controls, the underlying tinnitus abnormal brain regions can be discovered, which is helpful for finding the therapeutic targets of chronic tinnitus.

**Figure 2:**
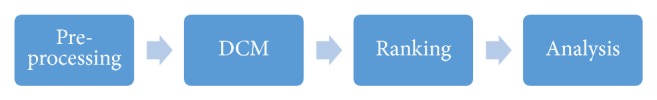
The main flowchart of the proposed method.

To this end, we study the steady-state EEG signals in the delta band (0.5-3.5Hz), theta band (3.5-7.5Hz), alpha band (7.5-12.5Hz), beta band (12.5-30Hz), and the gamma band (30Hz-45Hz) of 14 chronic tinnitus patients and 14 control individuals. By combining the dynamic causal modeling (DCM) and the exponential ranking algorithm, the insight of the abnormal brain regions of tinnitus can be gained, and the differences between tinnitus patients and the controls in neuronal aspects can be analyzed. For clarity, Figure 2 shows the main flowchart of our method.

## 2. Methods and Materials

### 2.1. Participants

Participants with chronic subjective tinnitus were recruited from the Ear, Nose and Throat (ENT) clinic, Sun Yat-sen Memorial Hospital, Sun Yat-sen University. Detailed selection criteria for inclusion in this study are as follows:

1. They had sought clinical help for their tinnitus problem, which had lasted more than 3 months.
2. They had no history of head trauma or central nervous system disorders.
3. They had mild sensorineural hearing loss. All tinnitus patients with either current conductive hearing loss or previous middle ear surgery (e.g., mastoidectomy) were excluded [26].
4. The tinnitus patients with pulsatile tinnitus due to aberrant vascular malformation and Menieres disease were also excluded.

This study included 14 patients (8 male and 6 female; age M=38.57 years, SD=13.91 years) with chronic tinnitus and 14 age and gender matched control subjects (6 male and 8 female; age M=38.71 years, SD=11.78 years). Only right-handed individuals were accepted for the study. All participants were comprehensively informed about the background and the aim of this study. And they all gave written informed consent. This study was approved by the ethics committee of Sun Yat-sen Memorial Hospital, Sun Yat-sen University.

### 2.2. EEG Recordings

All participants were required to calm down and sat on a comfortable chair in a completely silent room. They were instructed to open their eyes and fixate a cross mark on the computer screen. The recordings were made utilizing a dense array EEG system with 128 channels and were saved electronically with Electrical Geodesics, Inc. The sampling rate was set to 1000 Hz and impedances were kept below 50 k*ω*. The CZ electrode was used as reference for online recording. The resting EEG was recorded for about 5 minutes.

### 2.3. Preprocessing of EEG Data

Firstly, the raw data files from EGI were transformed into mat file format in order to preprocess them with EEGLAB for v13.0.0 toolbox of Matlab. Secondly, the sampling rate of the data was reduced to 250 Hz. The notch filter in ERPLAB was used to remove the 50 Hz power line interference. The data were band-pass-filtered to 0.5-80 Hz. Next, the reference electrode was removed and the bilateral mastoid 56th and 107th electrodes were rereferenced. Then, the 8th, 14th, 17th, 21st, 25th, 125th, 126th, 127th, and 128th electrodes were excluded from the EEG date, for they are either greatly affected by the eye movement or simply having too much noise in the detected data. Furthermore, all episodic artifacts including eye blinks, eye movements, teeth clenching, body movement, or ECG artifact were removed from the EEG waves via manual artifact rejection. Finally, independent component analysis (ICA) was applied to remove noise fragments.

### 2.4. Dynamic Causal Modeling

In the past several decades, many methods have been developed or adopted to extract information from biological signals. Biological signals like EEG and MEG are generated by biological systems and therefore are reflections of the nonlinear underlying activities. Classic feature extracting methods such as spectral analysis are only reasonable and explainable under assumptions that the system is stable, which means that the dynamics of the systems cannot be represented by the extracted features. So for systems like human brain, the classic feature extraction methods may not be suitable because they can neglect the hidden dynamics or causal effects. Dynamic causal modeling is a method to infer the causal architecture of distributed dynamic systems.

Notice that the statement that EEG data directly displays the cerebral neuronal activities of a subject is incorrect. In fact, it is the electromagnetic activity that are detected and named as EEG signals. The macrobiological meaning of EEG signals is that it is the electromagnetic responses to the neuronal activities caused by the wide-spread brain sources. So, the brain can be seen as a system that takes stimuli as input and outputs signals at a neuronal level and eventually follows the electromagnetic principles detected by sensors. Here, we are interested in the underlying neuronal dynamics. The dynamic causal modeling for M/EEG is a measure designed for such purpose. It is considered one of the best methods that could be applied to brain signals due to the following five reasons [27].

1. DCM is dynamic.
2. It is causal in the sense of control theory.
3. It makes efforts to interpret neurophysiology.
4. It uses a biophysically motivated and parameterized forward model to link the modeled neuronal dynamics to specific features of the measured data.
5. It is Bayesian in all aspects, which means that each parameter is constrained by a prior distribution.

**Figure 3:**
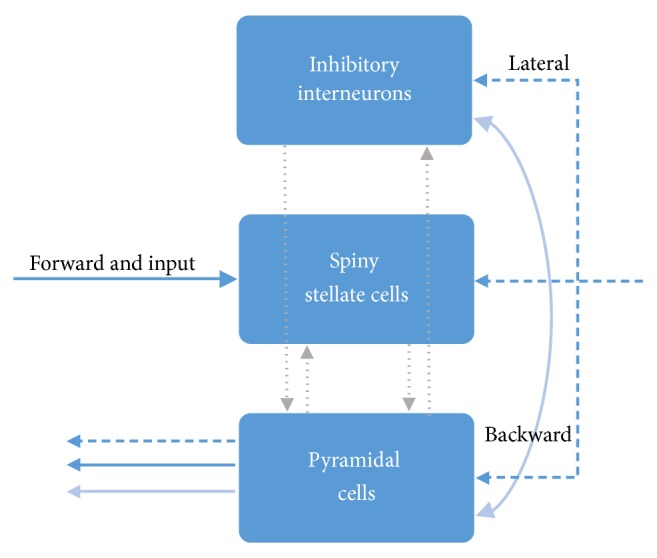
The neural mass model of a single source consists of 3 neuronal subpopulations, connected by 4 intrinsic connections (displayed by the grey spotted arrows) and 3 types of extrinsic connections [8].

Neuronal activities in a brain source are realized by three neuronal subpopulations as shown in Figure 3, namely, spiny stellate cells, pyramidal cells, and inhibitory interneurons. DCM for M/EEG adopts a neural mass model [28] to explain source activity in terms of the ensemble dynamics of interacting inhibitory and excitatory subpopulations of neurons. One can regard a brain source as a representative of the collective effects of its three subpopulations of neurons.

As shown in Figure 3, within a source, there are four intrinsic connections that transfer signals to each layer. And among these sources, there are three types of connections, namely, forward, lateral, and backward. Forward connections originate from the pyramidal cell subpopulation and end in spiny stellate cell subpopulation, while backward connections connect inhibitory interneuron subpopulation and pyramidal cell subpopulation, and lateral connections originate from the pyramid cell subpopulation and target at all three subpopulations. The input arrives at each subpopulation as mean firing rates through connections, while the output of each subpopulation is its membrane potential.

The values of the mean firing rates and membrane potential are parameterized by neuronal parameters listed in Table 1. Here we give a brief introduction of them. Readers can refer to the previous work [28] for more biological details of the neuronal mass model. *H*_*e*_, *H*_*i*_, *κ*_*e*_, *κ*_*i*_, *κ*_*a*_ are synaptic parameters, while *γ*_1_, *γ*_2_, *γ*_3_, *γ*_4_ are the intrinsic strengths and *ρ*_1_ and *ρ*_2_ are the sensitivity of the neural population to input and adaptation [29]. *κ* = 1/(*τ*) is a lumped rate constant of passive membrane and *H* represents the maximum postsynaptic potential. Inhibitory interneurons receive inputs from the pyramidal cells, which leads to excitatory postsynaptic potentials *H*_*e*_ mediated by the coupling strength between pyramid cells and inhibitory interneurons, *γ*_3_. The pyramidal cells also produce excitatory postsynaptic potentials mediated by coupling strength *γ*_2_, driven by excitatory spiny cells. The pyramidal cells provide inhibitory postsynaptic potentials mediated through parameter *γ*_4_, driven by the interneurons. Since the synaptic dynamics are linear, subpopulations are modeled with linearly separable synaptic responses to excitatory and inhibitory inputs.

A brain source is modeled by the aforementioned structure that explains the nonlinear behaviors. Since signals generated by a brain can be seen as a mixture of the responses to the inputs on these sources, we can then apply the general theory of linear system to the dynamics of EEG in forms of the neuronal mass model.

**Figure 4:**
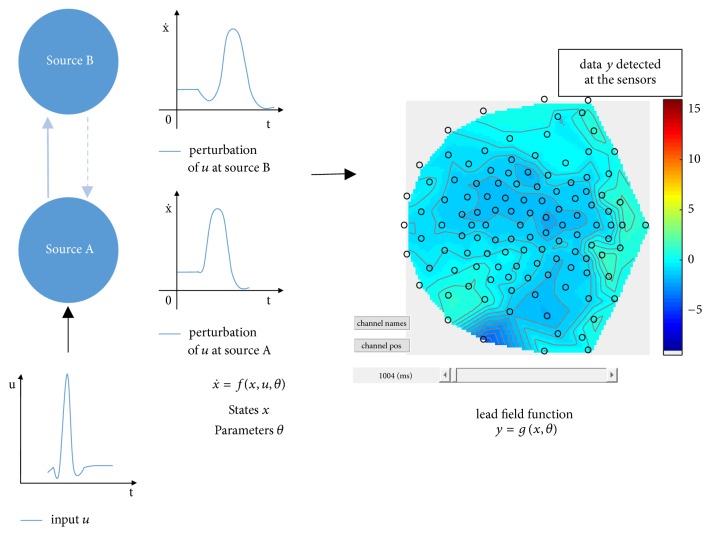
Illustration of a brain described by 2 sources, A and B, respectively. Inputs *u*, defined as a function of time *t*, are added to the brain system. The perturbation of the inputs at each source is described as a differential equations $\dot{x}=f(x,u,\theta )$, where *u* is the input and *θ* are the source parameters that vary along with experimental settings. How a source will be measured in the sensors is then described by the lead-field function *y* = *g*(*x*, *θ*) [8].

Take a study of two brain sources as an example, the underlying mechanism is shown in Figure 4:

- Deterministic inputs *u*(*t*), which can be seen as a function about time *t*, are added to the brain system.
- The pyramidal cells depolarize and cause responses in the brain sources. These responses are described as differential equations, as will be described in detail soon.
- The dynamic in each source can cause a signal in the sensors, at every moment in time. From the sensor perspective, the responses to neuronal activities are expressed in a lead-field function. A lead-field function is a function defining how responses on sources are measured in sensors, taking neuronal states *x* and neuronal parameters *θ* as parameters, as will be described in detail soon.

#### 2.4.1. Neuronal State Equations

The coupling relation of brain activity in different brain regions can be expressed as the neuronal state equations:

(1) $$\begin{array}{c} \dot{x}=f\left(\;x,u,\theta \;\right) \end{array}$$

where $\dot{x}$ represents the changes in neural activities, *f* is the nonlinear function describing the neurophysiological influences that activity *x* in all *l* brain regions, inputs *u* exert upon changes in the others, and *θ* are the parameters of the model whose posterior density is what we require for inference.

The bilinear approximation of (1) provides a set of equations in forms of the effective connectivity that can be calculated directly as follows:

(2) x˙=Ax+∑ujBjx+Cu=A+∑ujBjx+Cu

(3) A=∂f∂x=∂x˙∂x

(4) Bj=∂2f∂x∂uj=∂∂uj∂x˙∂x

(5) C=∂f∂u.

where matrix *A* is the effective connectivity representing the lateral connectivity among brain regions without input, *B*^*j*^ is the change in the coupling induced by the *j*-th input *u*_*j*_, and matrix *C* represents the extrinsic influences of inputs on neuronal activities.

Parameters *θ*^*c*^ are defined as *θ*^*c*^ = {*A*, *B*^*j*^, *C*}, which are the connectivity we wish to achieve to define the functional architecture and the interactions among brain regions of interest.

To spatially model the response, an equivalent current dipole for each source is specified. There are 16 regions of interest selected by visual inspection according to the previous work [30], as shown in Table 2. The 16 brain regions that we are interested in include two sides of superior and transverse temporal gyrus, two sides of cuneus/precuneus, middle occipital gyrus, precentral gyrus, superior frontal gyrus, prefrontal cortex, superior parietal cortex, basal ganglia/NAc, isthmus of cingulate gyrus, two sides of thalamus, brainstem, and Parahippocampal gyrus. Maudoux et al. [30] applied ICA to make sure the analyzed fMRI signal corresponds to the auditory spontaneous activity. The selection of the components of interest was based on another previous research which also takes advantage of ICA to decompose the signals in neuronal sources while preserving the concept of connectivity in a defined network of ROIs [31]. During the selection, to ensure that the components selected represent the auditory activity, they employed ROIs that were mentioned in a few papers on the auditory resting-state network.

#### 2.4.2. Electromagnetic Model

The neuronal activity affects the electromagnetic signals and causes the underlying changes. According to David et al. [32], the medical variables have nonlinear relationship between each other, i.e.,

(6) $$\begin{array}{c} y=g\left(\;x,\theta \;\right)=LKx_{0}. \end{array}$$

where *L* is a lead-field matrix representing the passive conduction of the electromagnetic field, and *K* is a matrix of *θ* that controls the contribution of pyramidal depolarization to the *i*-th source density.

According to the previous study [29], how responses of sources are measured in sensors can be expressed as functions of the neuronal parameters. After obtaining the equations of the two models, the EEG data can therefore be applied to the Bayesian estimation for inferences of the parameters.

#### 2.4.3. Estimation

For a given DCM model *m*, parameter estimation corresponds to approximating the moments of the posterior distribution given by Bayes rule as follows:

(7) $$\begin{array}{c} p\left(\;\theta \;|\;y,m\;\right)=\frac{p\left(y\;|\;\theta ,m\right)p\left(\theta ,m\right)}{p\left(y\;|\;m\right)}. \end{array}$$

Bayesian inference proceeds using the conditional or posterior density estimated by iterating (7). This is an expectation maximization method, which solves the special maximum likelihood problems in an iterative way.

The posterior moments including the mean *μ* and covariance ∑ are estimated iteratively using Gaussian approximation to the conditional density *q*(*θ*) = *N*(*μ*, ∑). The basic idea behind expectation maximization is that we calculate the expectation of the posterior probability and then use the maximum likelihood method to infer the parameters. Chen et al. [33] have summarized the estimation scheme as follows.

while (unconvergence)

E-step *q* ← max_*q*_(*F*(*q*, *λ*, *m*))

M-step *λ* ← max_*λ*_(*F*(*q*, *λ*, *m*)

*F*(*q*, *λ*, *m*) = 〈ln⁡*p*(*y*∣*θ*, *λ*, *m*) − ln⁡*p*(*θ*∣*m*)〉_*q*_

*F* is the variational free energy approximating to the posterior density *p*(*θ*∣*y*, *m*) which we require. And *λ* are the precision parameters that are updated to estimate the maximum likelihood in the previous step. The expression 〈·〉_*q*_ means the expectation under the density *q*.

For implementation of DCM, the open-source software within the Statistical Parametric Mapping (SPM) software is used (http://www.fil.ion.ucl.ac.uk/spm/).

### 2.5. Exponential Ranking

Since there are two groups of subjects (i.e., the tinnitus group and the control group) to be compared, the dynamic causal models described in the previous section should be the same. This leads to a negative connectivity meaning that the estimation is far smaller than the prior 1. In order to get the “reputation” (i.e., the ranking) of a brain source in the network, we need to choose a PageRank-like algorithm applicable to signed networks. The exponential ranking [25] designed in 2010 meets this need, which is based on the discrete choice theory. The basic idea is that if a node has negative reputation, his links should be trusted less instead of not trusted. Most of the existing algorithms dealing with negative links do not apply distrust in such recursive manner and if so, they simply take away nodes that have negative links. In this section, we will give a brief description of the exponential ranking algorithm.

Let *G* = (*V*, *E*) be a directed graph with *n* nodes in *V* and *m* edges in *E*. The edges are represented as an *n* × *n* adjacency matrix, where *A*_*ij*_ is 0, 1 or -1, meaning the edge linked from node *i* to node *j*. In particular, a negative link between node *i* and *j* means that *i* distrusts *j*.

The reputation of node *i*, denoted as *k*_*i*_, is calculated as follows:

(8) $$\begin{array}{c} k_{i}=\underset{j}{\sum }A_{ji}p_{j} \end{array}$$

where *p*_*i*_ is the trust probability of node *i* and is calculated as follows:

(9) $$\begin{array}{c} p_{i}=\frac{\exp\left(k_{i}/\mu \right)}{\sum_{j}^{}\exp\left(k_{j}/\mu \right)} \end{array}$$

where *μ* characterizes the amount of noise. In the matrix notation, (8) can be rewritten as

(10) $$\begin{array}{c} k=A^{T}p \end{array}$$

Intuitively, if one has to choose a node to trust, he may choose the one with the highest reputation *k*. However, there might be some errors while just considering the reputation. To this end, the error caused by noise should be considered and denoted by *μ*. Please refer to the related paper [25] for more detail.

Combining the two equations, we obtain the recursive formulation as follows:

(11) $$\begin{array}{c} p\left(\;t+1\;\right)=\frac{\exp\left(1/\mu \right)A^{T}p\left(t\right)}{{\left\|\exp\left(1/\mu \right)A^{T}p\left(t\right)\right\|}_{1}}. \end{array}$$

For clarity, Algorithm 1 summarizes the main procedure of the exponential ranking algorithm.

## 3. Results

To investigate the performance of different response time, we repeated the experiment by setting the response time as 100ms and 2000ms. It turns out that the largest difference between the mean rankings of the tinnitus group and the control group can be obtained when setting the response time as 60ms, which can be further confirmed by Table 3. In Table 3, for each type of response time (e.g., 60ms), the variance of the absolute differences between the two mean ranking values of the tinnitus group and the control group is calculated for each frequency band, respectively, and then the mean value of the five variances is reported in the table.

**Figure 5:**
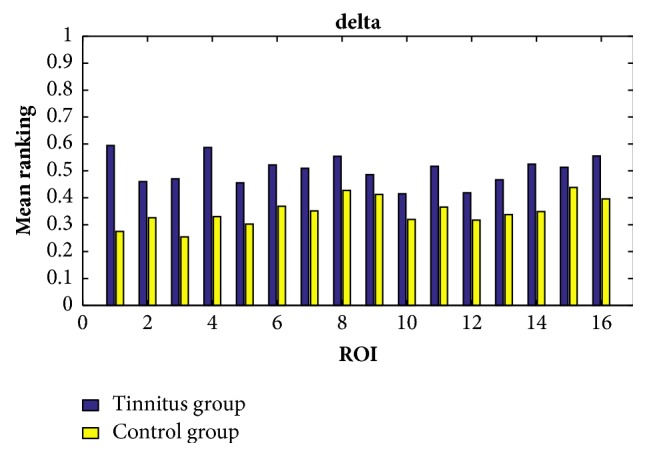
The ranking values calculated from the functional connectivity in the delta band of controls (yellow) and tinnitus (blue) patients by setting the response time as 60ms.

**Figure 6:**
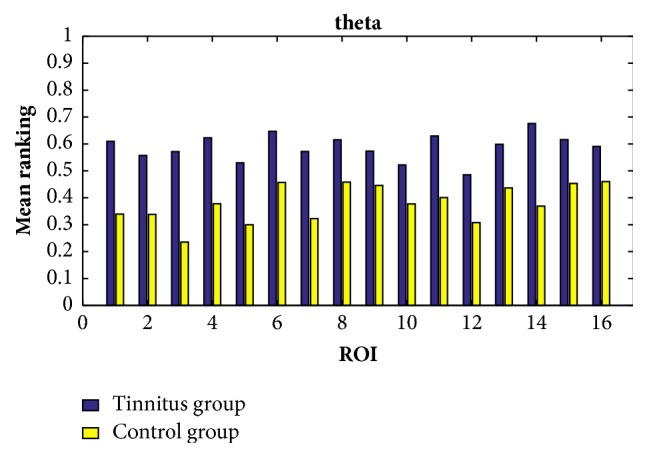
The ranking values calculated from the functional connectivity in the theta band of controls (yellow) and tinnitus (blue) patients by setting the response time as 60ms.

**Figure 7:**
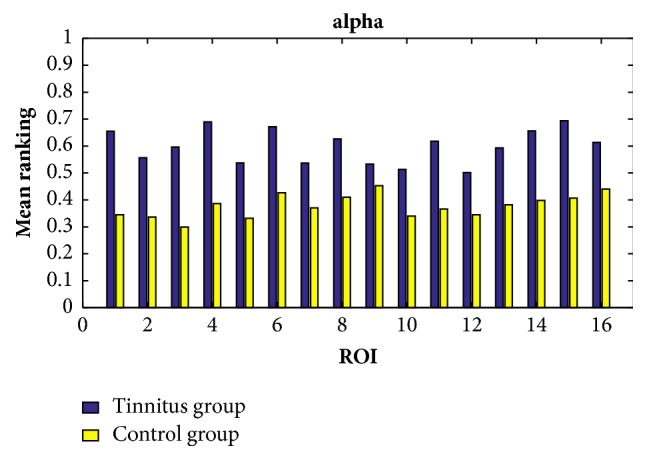
The ranking values calculated from the functional connectivity in the alpha band of controls (yellow) and tinnitus (blue) patients by setting the response time as 60ms.

**Figure 8:**
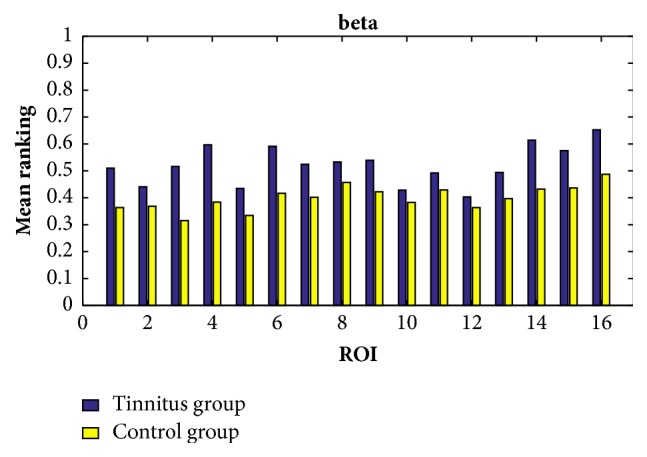
The ranking values calculated from the functional connectivity in the beta band of controls (yellow) and tinnitus (blue) patients by setting the response time as 60ms.

**Figure 9:**
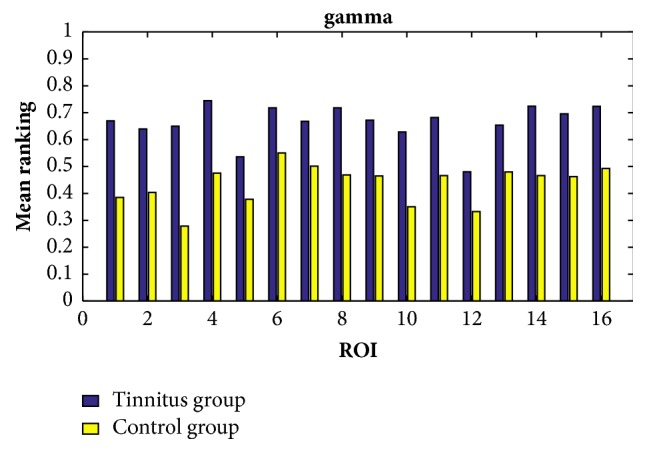
The ranking values calculated from the functional connectivity in the gamma band of controls (yellow) and tinnitus (blue) patients by setting the response time as 60ms.

In order to find the common characteristics within each group, we consider the average ranking values among all the participants within the same group in all the cases of the delta, theta, alpha, beta, and gamma bands. By setting the response time as 60ms, the exponential ranking of the tinnitus group and the control group obtained from the functional connectivities in delta, theta, alpha, beta, and gamma bands is shown in Figures 5, 6, 7, 8, and 9, respectively. All the ranking results range from 0 to 1, with the value representing the reputation of the very brain source among all the 16 brain regions. The order of ROIs does not necessarily have any meaning, and we focus on the relative differences of the two groups rather than the absolute differences.

Overall, the chronic tinnitus patients, compared to the normal controls, have shown increased functional connectivity in the right superior and transverse temporal gyrus (the 1st ROI) and left cuneus/precuneus (the 4th ROI) in all frequency bands, and in the right cuneus/precuneus (the 3rd) and brainstem (the 14th ROI) in all frequency brands except the theta band, and in middle occipital gyrus (the 5th ROI) in all frequency brands except the gamma band.

Another interesting finding is that the importance of the two sides of superior and transverse temporal gyrus (the 1st and 2nd ROI) is greatly different in tinnitus patients, with the right side showing an increased connectivity. And the right side of cuneus/precuneus (the 3rd ROI) is also having more activated signals comparing to the left side.

## 4. Discussion

In this study, we combined DCM and exponential ranking to detect the abnormal brain regions in tinnitus patients comparing with healthy controls. Present study found increased intrinsic connectivity in the parietal and prefrontal cortices, nucleus accumbens (NAc), isthmus of cingulate gyrus, thalamus, and brainstem.

The intrinsic connectivity in the parietal and prefrontal cortices was significantly increased in tinnitus patients. Kleinjung et al. [34] found that repetitive transcranial magnetic stimulation (rTMS) of the temporal and prefrontal cortices showed a better long-term effect in the tinnitus patients than rTMS of the temporal cortex. It indicated that the prefrontal cortex was involved in the pathophysiology of tinnitus. Moreover, the prefrontal cortex was considered as an area to integrate the cognitive and emotional aspects of tinnitus [35, 36]. The prefrontal cortex was involved in attention control and the increase of the intrinsic connectivity in the prefrontal cortex was in line with the hypothesis that tinnitus might be related to the excessive allocation of attention [30]. In addition, according to the study of Vanneste et al. [37], the dorsolateral prefrontal cortex was related to the distress of tinnitus patients. Therefore, as the increase of attention and emotion to the tinnitus, the intrinsic connectivity in the parietal and prefrontal cortices was raised.

The activation of nucleus accumbens (NAc) in this study was consistent with the study of Leaver et al. [38]. According to the model proposed by Rauschecker et al. [17], the tinnitus signals could be tuned out at the level of the thalamus by the feedback connection from limbic system, which prevents the tinnitus signals from reaching the auditory cortex. The nucleus accumbens as a part of the limbic system was abnormal in tinnitus patients, which influenced the cancellation of tinnitus signals and led to the perception of tinnitus.

The isthmus of cingulate gyrus had the function of contacting the left and right hemispheres. The increased connection of the isthmus of cingulate gyrus in tinnitus patients indicated the increased relationship between the left and right brain hemispheres.

The auditory system includes the auditory cortex, the inferior colliculus in the thalamus, and the cochlear nucleus in the brainstem. Tinnitus patients with peripheral hearing loss showed decreased signal output from the cochlea, which led to the loss of some auditory information. As a result of deafferentation, activity in some specific brain areas of the auditory pathway declines and the regions near the specific brain areas show increased activity due to the decrease of lateral inhibition. So the missing auditory information is retrieved from the adjacent auditory pathway [39, 40]. Therefore, the deficit might be compensated for at the level of the thalamus or brainstem to maintain homeostatic state and reduce the uncertainty, which may be the reason why the intrinsic connectivity in the thalamus and brainstem was significantly increased.

Additionally, the tinnitus patients are found to have an increased extrinsic connectivity in cuneus/precuneus, middle occipital gyrus, and precentral gyrus. It represented the increased extrinsic connections of the visual and somatomotor networks. Cacace [41] suggested that tinnitus was triggered or modulated by inputs from somatomotor and visual systems in a proportion of individuals.

The cuneus/precuneus have a higher lateral connectivity in this study. Some fMRI studies showed that there is a negative correlation between auditory and visual resting-state networks in tinnitus patients [30, 42]. It may decrease the activity in visual network due to the increase of attention to the tinnitus perception. Therefore, tinnitus may be considered as a result of a multisensory interaction between the auditory and visual networks [43].

Furthermore, tinnitus patients showed a stronger connection in the right side than the left side of superior and transverse temporal gyrus. The right side of cuneus/precuneus was also having more activated signals comparing to the left side. It suggested that the right side of the auditory and visual cortices was more active than the left side. This result implied the difference of the connectivity between the left and right sides of the auditory and visual systems in tinnitus patients.

## 5. Conclusions

In conclusion, this is the first study to explore the abnormal brain regions by the combination of DCM and exponential ranking. Tinnitus patients showed increased intrinsic connectivity in the parietal and prefrontal cortices, nucleus accumbens (NAc), isthmus of cingulate gyrus, thalamus, and brainstem, which associated with the perception, emotion, and attention of tinnitus. In addition, the extrinsic connectivity to the visual and somatomotor systems was improved in tinnitus patients, which suggested the important role of the visual and somatomotor systems in tinnitus. Furthermore, this study indicated that DCM, combined with exponential ranking, was a helpful approach to detect the abnormal brain regions and was beneficial for the diagnosis and treatment of tinnitus.

## Figures and Tables

**Algorithm 1:**
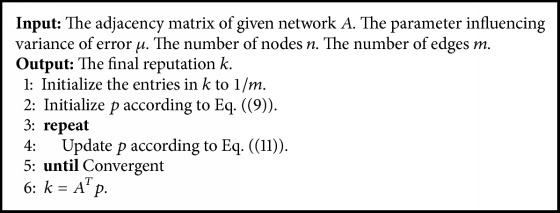
Exponential ranking.

**Table 1:** Electromagnetic parameters.

Parameter	Description	Standard prior mean
*H*_(*e*/*i*)_	Maximum post synaptic potentials	4 mV, 32 mV
*τ*_(*e*/*i*)_ = 1/*κ*_(*e*/*i*)_	Average dendritic and membrane rate constant	4 ms, 16 ms
*τ*_(*e*/*i*)_ = 1/*κ*_*a*_	Adaption rate constant	512 ms
*γ*_(1,2,3,4)_	Average number of synaptic contacts among populations	128, 128, 64, 64, 16
*ρ*_1_, *ρ*_2_	Parameterized gain function *g*	2, 1

**Table 2:** The 16 regions of interest.

Brain region (area)	x	y	z
1. Superior & transverse temporal gyrus (R)	62	-18	23
2. Superior & transverse temporal gyrus (L)	-50	-15	11
3. Cuneus/Precuneus (19/31) (R)	9	-64	25
4. Cuneus/Precuneus (19/31) (L)	-15	-64	25
5. Middle occipital gyrus (L)	-45	-52	7
6. Precentral gyrus (L)	-33	-19	46
7. Superior frontal gyrus (R)	6	5	46
8. Prefrontal cortex (L)	3	47	16
9. Superior parietal cortex (R)	54	-22	52
10. Basal ganglia/NAc (R)	15	-1	-5
11. Isthmus of Cingulate Gyrus (L)	-9	-40	1
12. Thalamus (R)	9	-13	10
13. Thalamus (L)	-15	-19	-2
14. Brainstem (R)	6	-19	-23
15. Parahippocampal gyrus (L)	-21	-28	-27
16. Parahippocampal gyrus (R)	27	-25	-14

**Table 3:** The average variance of the meaning ranking between the tinnitus group and the control group when setting different response time.

Response time	Average variance
60ms	0.0582
100ms	0.0407
2000ms	0.0485

## Data Availability

No additional data are available. However, the original data that support the findings derived from this study can be requested by emailing panada810456@126.com.
